# A novel testis-enriched gene, *Samd4a*, regulates spermatogenesis as a spermatid-specific factor

**DOI:** 10.3389/fcell.2022.978343

**Published:** 2022-10-05

**Authors:** Jinsoo Ahn, Dong-Hwan Kim, Mi-Ryung Park, Yeunsu Suh, Haesun Lee, Seongsoo Hwang, Lovelia L. Mamuad, Sang Suk Lee, Kichoon Lee

**Affiliations:** ^1^ Department of Animal Sciences, The Ohio State University, Columbus, OH, United States; ^2^ Animal Biotechnology Division, National Institute of Animal Science, Wanju, South Korea; ^3^ Department of Animal Science and Technology, Sunchon National University, Suncheon, South Korea

**Keywords:** *Samd4a*, testis-enriched genes, knockout mice, spermatogenesis, non-obstructive azoospermia

## Abstract

Spermatogenesis is the highly orchestrated process involving expression of a series of testicular genes. Testis-enriched genes are critical for cellular processes during spermatogenesis whose disruption leads to impaired spermatogenesis and male infertility. Nevertheless, among poorly investigated testicular genes are the mouse *Samd4a* and human *SAMD4A* which were identified in the current study as novel testis-enriched genes through transcriptomic analyses. In particular, as orthologous alternative splicing isoforms, mouse *Samd4a* E-form and human *SAMD4A*C-form containing the SAM domain were specific to testes. Western blot analyses revealed that the murine *SAMD4A*E-form was predominantly found in the testis. Analyses on GEO2R and single-cell RNA-seq datasets revealed that the *Samd4a*/*SAMD4A* expression was enriched in spermatids among various types of cells in adult testes. To investigate *in vivo* functions of *Samd4a*, *Samd4a* knockout mice were generated using the CRISPR/Cas9 system. The *Samd4a* deficiency resulted in lower testis weight, absence of elongated spermatids, and an increased number of apoptotic cells. Profiling of gene expression in human testis samples revealed that the *SAMD4A* expression was comparable between obstructive azoospermia patients and normal controls, but significantly lowered in nonobstructive azoospermia (NOA) patients. Among three subgroups of NOA, pre-meiotic arrest (NOA-pre), meiotic arrest (NOA-mei), and post-meiotic arrest (NOA-post), expression level of *SAMD4A* was higher in the NOA-post than the NOA-mei, but there was no difference between the NOA-pre and NOA-mei. The current studies demonstrated spermatid stage-specific expression of *Samd4a*/*SAMD4A*, and impairment of the late stages of spermatogenesis by disruption of the mouse *Samd4a* gene. These data suggest that *Samd4a*/*SAMD4A* plays an essential role in normal spermatogenesis, and SAMD4A, as a spermatid specific marker, can be used for subcategorizing NOA patients. Further understanding the molecular role of SAMD4A will advance our knowledge on genetic regulations in male infertility.

## Introduction

Spermatogenesis is the highly orchestrated process involving the periodical expression of a series of testicular genes in a spatiotemporal manner at various stages of spermatogonia, spermatocytes, and spermatids (Nishimune and Tanaka, 2006). Genes expressed specifically in testis constitute the largest number of tissue-enriched genes with more than 1,000 genes, while it has been estimated that there are approximately 290 tissue-specific genes on average (Yu et al., 2006; Djureinovic et al., 2014; Uhlen et al., 2015). As tissue-enriched genes play fundamental roles in regulating development of tissues and organisms, differential expression of testis-enriched genes is critical for cell proliferation, differentiation, RNA binding for translational regulation, meiotic division, chromatin condensation, and spermiogenesis (Tanaka and Baba, 2005; Djureinovic et al., 2014). Based on developmental stage-specific expression of testis-enriched genes, single-cell RNA-seq has greatly advanced transcriptomic analyses in terms of defining testicular cell types throughout development *via* unsupervised cell clustering (Guo et al., 2018; Hermann et al., 2018). However, as previously noted in studies (Djureinovic et al., 2014; Chen et al., 2021), there are still a substantial portion of genes and gene transcripts that are expressed in each developmental stage of the testis, but poorly investigated.

In our previous comparative studies, based on transcriptomic analyses combined with confirmatory gene and protein expression profiling, testis enrichment of 24 common genes in mice and humans and their subcellular localization were revealed (Ahn et al., 2017). During extension of these investigations on testis-enriched genes, a previously unreported testis-specific gene, sterile alpha motif domain containing 4 (*Samd4*/*SAMD4*), aka *Samd4a*/*SAMD4A*, that encodes several alternative splicing transcript isoforms was identified. SAMD4A is an RNA-binding protein involved in translational repression operates through the sterile alpha motifs (SAM) domain that binds to Smaug recognition elements (SRE) in mRNAs (Aviv et al., 2003; Baez and Boccaccio, 2005). It has been reported that *SAMD4A* formed cytoplasmic granules containing polyadenylated mRNA and *SAMD4A* knockdown led to impaired cytosolic membraneless organelle (MLO) condensation which released unrepressed mRNAs (Baez and Boccaccio, 2005; Fernandez-Alvarez et al., 2022).

In abnormal conditions such as in cancer drug-resistant cell lines and cardiomyocytes upon myocardial infarction, dysregulated expressions of *SAMD4* and its related noncoding transcript, respectively, have been reported (Klejewski et al., 2017; Zheng et al., 2022). In addition, *Samd4*-deficient mice exhibited delayed bone development and impaired osteogenesis (Niu et al., 2017). Moreover, mice carrying a homozygous *Samd4-*missense mutation showed leanness, myopathy, and adipocyte defects, and these mice were reported to be infertile (Chen et al., 2014). However, there is a lack of further investigation of effects of *Samd4* depletion on infertility. Herein, we hypothesized that the testis-enriched *Samd4a/SAMD4A* containing the SAM domain play crucial roles especially in testes, and the timely regulation of this testicular gene will be implicated in normal spermatogenesis.

Therefore, by means of transcriptomic analyses and RT-PCR, we examined testis- and developmental stage-enrichment and alternative splicing isoforms of *Samd4a/SAMD4A* in mice and humans. Subsequently, the current study focused on investigating effects of *Samd4a* deficiency on spermatogenesis for the first time by generating *Samd4a* knockout mice. To further relate *SAMD4A* to male reproduction, the *SAMD4A* expression was profiled using data from non-obstructive and obstructive azoospermia patients.

## Materials and methods

### Study design, animal use and sample preparation

To analyze gene and protein expression patterns of *Samd4A*/*SAMD4A* in various mice tissues, RT-PCR and Western blot analysis were conducted (at least triplicates for each tissue). The effects of gene knockout were investigated after generating *Samd4a* knockout mice as described below and by analyzing testes samples (*n* = 5). All animal care and procedures were approved by the Institutional Animal Care and Use Committees (IACUCs) of Sunchon National University (protocol number: SCNU IACUC-2022–10) and The Ohio State University (protocol number: 2007A0183). Mice were raised under *ad libitum* feeding conditions in mice housing facilities before euthanization *via* carbon dioxide inhalation followed by cervical dislocation. For isolation of murine total RNAs, the testis, muscle, liver, brain, lung, kidney, adipose tissue, heart, spleen, and small intestine were collected from three-month-old FVB mice using Trizol reagent (Invitrogen, Carlsbad, CA, United States) as described previously (Ahn et al., 2018). Total RNAs from the adult human testis, muscle, liver, brain, lung, kidney, thymus, and heart were purchased from Agilent Technologies (Santa Clara, CA, United States) and adult human RNA from adipose tissue was purchased from Clontech Laboratories (Mountain View, CA, United States). Three-month-old C57BL/6 mice were euthanized for protein extraction from the same tissues as described in our reports (Ahn et al., 2018).

### Generation of *Samd4a* knockout mice

CRISPR guide RNA (gRNA) (5′ - CTG​ACG​ATA​AGC​TCA​ATG​GC 3′) was designed to target mouse *Samd4a* exon three of A-through C-forms and exon two of D- and E-forms. Restriction by the gRNA and Cas9 protein was validated *in vitro* by incubation of the reaction mixture including gRNA, Cas9 protein, substrates, 10x BSA, and buffer at 37°C for 1 h 30 min, addition of RNase and incubation at 37°C for 20 min, and addition of stop solution and incubation at 37°C for 20 min, followed by separation on a 2% agarose gel. The gRNA was microinjected into 1-cell stage mouse embryos. Twenty-nine pups were born and genotyped by PCR using a primer set (forward: 5ʹ -ACC​GAT​CCC​ATC​CAC​AGT​TGA - 3ʹ and reverse: 5ʹ - ACC​AAA​CCC​GAA​TGC​GTT​CCA - 3ʹ). This genotyping and subsequent sequencing revealed that four F_0_ founders had eight bp deletion which gave rise to frameshift mutation. Those four F_0_ founders were mated to produce heterozygous and homozygous *Samd4* knockout mice. Sequencing analysis of the litters was conducted for the target site to confirm the heterozygous and homozygous mutant mice.

### Analyses of RNA-sequencing

For analyses of bulk RNA-sequencing (RNA-seq), raw data were retrieved from public databases and processing was performed as described previously in our report (Ahn et al., 2021). In detail, the raw FASTQ files were checked with FastQC v0.11.7 and then adapter-trimmed and filtered by Trimmomatic v0.38 with default parameters (Bolger et al., 2014). Cleaned reads were quantified against decoy-aware mouse and human transcriptome using Salmon v1.3.0 through the mapping-based mode (Patro et al., 2017). The gene expression values in transcripts per million (TPM) were collected from the Salmon output files (quant.sf) and imported into the R software v4.1.3 for boxplot visualization.

### Reverse transcription PCR (RT-PCR)

To measure the quantity of RNA, a Nanodrop spectrophotometer (Thermo Scientific, Wilmington, DE) was used. The RNA samples were stored at -80 °C until use. Approximately 1 µg of RNA was reverse-transcribed to cDNA in a 20 µl total reaction using Moloney murine leukemia virus (M-MLV) reverse transcriptase (Invitrogen). The thermal cycle of the reverse transcription was 65°C for 5 min, 37°C for 52 min, and 70°C for 15 min. Exactly 1 µl of cDNA samples was used as templates for PCR in a 25 µl total reaction with AmpliTaq Gold DNA polymerase (Applied Biosystems, Carlsbad, CA, United States). The conditions for this reaction were 95°C for 1 min 30 s 33 cycles of 94°C for 30 s, 55°C for 1 min, 72°C for 1 min, with an additional extension step at 72°C for 10 min. PCR products were separated by using 1% agarose gel electrophoresis. Forward and reverse primers for amplifying murine *Samd4a* and human *SAMD4A* transcripts are listed in Supplementary Table S1. DNAs extracted from the gel were sequenced by The Ohio State University Sequencing Core Facility using an Applied Biosystems 3730 DNA analyzer (Foster City, CA, United States).

### Overexpression of *Samd4a* C- and E-forms and Western blot analysis

The construction of expression vectors containing murine *Samd4a* isoforms was conducted as follows. Murine *Samd4a* C- and E-forms were amplified using sets of forward (m-h-*SAMD4A*/F3-full and m*Samd4a*/F2, respectively) and reverse (m-h-*SAMD4A*/R1) primers (Supplementary Table S1). Both isoforms were then cloned into a TA cloning vector, pCR2.1 (Invitrogen). After digestion of the cloning vectors with BamHI and NotI, the two fragments of C- and E-forms were ligated into an expression vector, pcDNA3.1 (Invitrogen) *via* the same enzyme sites. The expression vectors were transfected into HEK293 cells to overexpress the C- and E-forms using lipofectamine 2000 (Invitrogen) and collect positive protein controls 2 days after transfection. Western blotting was conducted as described in our previous report (Ahn et al., 2018). In detail, protein extracts from mouse tissues and positive controls (C- and E-forms) were separated on 12% SDS-PAGE and transferred to polyvinylidene difluoride (PVDF) membranes (Amersham Biosciences Hybond-P; Amersham Biosciences, Piscataway, NJ, United States). The membranes were blocked for 30 min in 4% nonfat dry milk in 1× Tris-buffered saline containing 0.05% Tween-20 (TBST) and then incubated overnight at 4°C with a rabbit polyclonal SAMD4A primary antibody raised against the C-terminal end (HPA065309, Atlas antibodies, Stockholm, Sweden). Next day, the membranes were washed with 1×TBST and incubated with the corresponding HRP-conjugated secondary antibody (rabbit IgG; HAF 008, R&D Systems, Minneapolis, MN, United States) for 1 h at room temperature. After washing with 1×TBST, Amersham ECL plus Western Blotting Detection Reagents (GE Healthcare Biosciences, Pittsburgh, PA, United States) was applied on the membrane, and the blots were developed onto Hyperfilm (GE Healthcare Biosciences).

### Analyses of single-cell transcriptome

For analyses of developmental stages of testicular cells, original BAM files generated by 10x Genomics platform and submitted to the NCBI SRA were retrieved from Amazon Web Service (AWS) (Accession number GSE109033 for the mouse; GSE109037 for the human). These single-cell RNA-seq (scRNA-seq) data from testicular cells were converted to FASTQ files using the bamtofastq utility (v1.3.1) in order to proceed with the 10x Genomics’ Cell Ranger pipeline. The cellranger count was used to align sequencing reads to the reference transcriptome (mm10 and GRCh38) for each sample. Using the cellranger aggr function, the outputs of the cellranger count for individual samples were combined with batch correction. These processed data were imported to the R package Seurat v4.1.0 for data normalization and unsupervised cell clustering, and UMAP analyses were performed based on statistically significant principal components (Satija et al., 2015; Hao et al., 2021). After identification of cell clusters based on the top 10 differentially expressed genes and stage-specific marker genes listed previously (Hermann et al., 2018), raw count matrices were imported to Monocle3 v1.0.0 (Trapnell et al., 2014; Cao et al., 2019) for dynamic trajectory analyses which aligned cells in pseudotime. The numbers of cells analyzed in this study are listed in Supplementary Table S2.

### Histological processing and TUNEL assay

After harvesting and photographing testes and measuring weights, the whole testes of wild-types, heterozygous *Samd4a* knockouts, and homozygous *Samd4a* knockouts were processed for histological analysis. In particular, testes were fixed and embedded in paraffin followed by sectioning into 5 µm slices. After deparaffinization, the testicular tissue sections were subjected to hematoxylin and eosin (H and E) staining to examine morphological changes of testicular cells in the seminiferous tubules. Periodic Acid-Schiff Reaction (PAS) stain for histochemical assay of the testes was processed for determining normal and abnormal stages of the seminiferous epithelia cycle. The terminal deoxyribonucleotidyl transferase-mediated dUTP nick-end labeling (TUNEL) assay was applied to detect apoptotic cells in testes. Photographing was performed using a camera (SM-N920L, Samsung, South Korea). Microscopic images of stained slides were captured by an imaging system, DP73 from Olympus or AxioCam MRc5 from Zeiss.

### Statistical analyses

To test the normality of the data, the Shapiro-Wilk normality test was conducted with log-transformed or non-transformed expression values. For non-normal data, the non-parametric Kruskal–Wallis test was performed followed by the Dunn’s post-hoc test. For normal data, one-way ANOVA was conducted followed by the Tukey’s post-hoc test. Student’s t-test in R was used to compare two means. The minimum level of significance was set as *p* < 0.05. All statistical tests were performed using R packages, stats v4.1.3 and FSA v0.9.3.

## Results

### Testis-enriched expression of murine *Samd4a* transcripts was identified at both gene and protein levels

**FIGURE 1:**
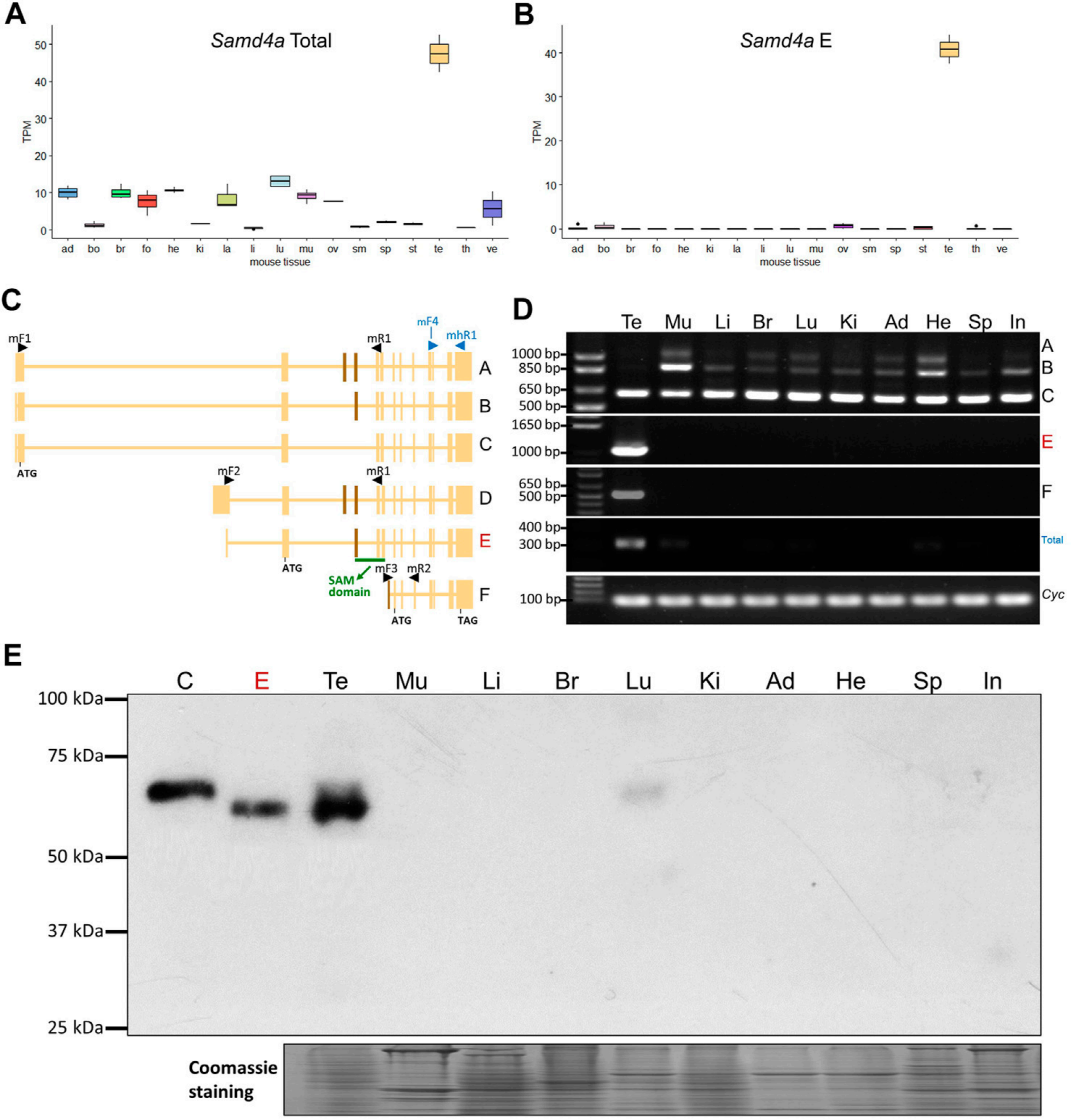
Expression profiling of murine *Samd4a*. **(A)** The total mRNA expression level of *Samd4a* transcripts in various mouse tissues based on RNA-seq (PRJNA375882). Ad, adrenal gland; bo, bone marrow; br, brain; fo, forestomach; he, heart; ki, kidney; la, large intestine; li, liver; lu, lung; mu, muscle; ov, ovary; sm, small intestine; sp, spleen; st, stomach; te, testis; th, thymus; ve, vesicular gland. **(B)** Among the total mRNA expression level, the mRNA expression level of the major *Samd4a* transcript (E form) is shown. **(C)** A schematic diagram of *Samd4a* transcripts based on the NCBI Gene database (https://www.ncbi.nlm.nih.gov/gene/74480). Black arrow heads indicate forward and reverse primers for RT-PCR. Blue arrow heads denote primers for total transcripts. Non-overlapping exons are indicated with brown perpendicular bars. Exons corresponding to the SAM domain in the translated protein is underscored in green. **(D)** Alternative splicing variants of murine *Samd4a* amplified by RT-PCR. Total, total transcripts. The cyclophilin (*Cyc*) gene was used as a loading control. **(E)** Western blot analysis for murine SAMD4A C- and E-forms. C and E represent overexpressed C- and E-form protein controls in HEK293 cells. Te, testis; Mu, muscle; Li, liver; Br, brain; Lu, lung; Ki, kidney; Ad, adipose tissue; He, heart; Sp, spleen; In, small intestine.

Analyses of RNA-seq data revealed that the total expression level of *Samd4a* in the murine testis (∼47.5 TPM) was markedly high with an approximately 8.4-fold higher expression compared to an average TPM (∼5.7) of the rest of tissues (Figure 1A). Among analyzed transcript isoforms, one isoform (NCBI RefSeq accession: NM_001163433.1), *E-form*, was identified as testis-specific with an approximately 350-fold higher expression in the murine testis compared to an average TPM of the rest of tissues (Figure 1B), unlike other isoforms with variable expression levels in several tissues (Supplementary Figure S1). Using semiquantitative RT-PCR, transcripts were amplified to verify alternative splicing with four primer sets, mF1-mR1, mF2-mR1, mF3-mR2, and mF4-mhR1 (Figure 1C). As a result, with the first primer set (mF1-mR1), expression of *Samd4a C-form* was detected in all analyzed tissues including the testis, and the A- and B-forms were not observed in the testis (Figure 1D). On the other hand, using the second primer set (mF2-mR1), *Samd4a E-form* (1,065 bp) was detected only in the testis which contains the sterile alpha motif (SAM) domain, and the D-form (1,215 bp) was deficient in all analyzed tissues (Figure 1D). The *F-form* amplified using the third primer set (mF3-mR2) and the total transcript amplified using the fourth set of primers (mF4-mhR1) appeared to be upregulated in the testis (Figure 1D).

To further investigate expression patterns of *Samd4a* isoforms in different mouse tissues, Western blot analysis was performed including over-expressed *Samd4a* C- and E-forms. The *Samd4a* E-form protein (approx. 67 kDa, 610 amino acid residues) was predominantly expressed in the murine testis, and the C-form (approx. 69 kDa, 623 amino acid residues) was barely detectable in the testis and lung (Figure 1E). Other murine isoforms [A-form (approx. 84 kDa, 761 amino acid residues), B-form (approx. 78 kDa, 711 amino acid residues) and F-form (approx. 33 kDa, 302 amino acid residues)] were not detected. The murine E-form contains an intact SAM domain (63 amino acid residues, NCBI accession: cd09557) which binds to the Smaug recognition element in mRNAs, but the C-form lacks eight amino acids in the beginning of the SAM domain due to skipping of the exon corresponding to the exon three of the E-form (Figure 1C). In the human, the *SAMD4A* C-form protein (approx. 68 kDa, 617 amino acid residues) contains the 100% homologous SAM domain (SupplementaryFigure S2). Our data clearly showed that E-form mainly expressing in testis is predominant among different isoforms of *Samd4A* in mice.

### The orthologous *SAMD4A* mRNA in the human was enriched in the testis

Our analyses of GTEx dataset resulted in a median-based identification of the testis as the first-ranked tissue of *SAMD4A* expression among 47 human tissues with sample sizes ranged from 35 to 560 (Supplementary Figure S2A). Within reproductive human tissues, including the epididymis (caput, corpus, and cauda), and testis, expression levels of TPM of both the total and C-form were 9.6-fold and 21.3-fold higher in the testis compared to averages of the rest of reproductive tissues, respectively (Supplementary Figure S2B,C). The C-form in the human containing the full length of SAM domain (Supplementary Figure S2D) can be an orthologous alternative splicing isoform of the mouse *Samd4a* E-form. The data from RT-PCR with four different primer sets for the human (hereafter, parenthesized with abbreviations of forward and reverse primers) showed ubiquitous expression of A- and B-forms (mhF3-hR1), predominant C-form expression in the testis (hF4-hR1), as well as, testicular expression of D-form (hF4-hR1), E-form (hF2-hR1), and the total transcript (hRealF1-mhR1) (Supplementary Figure S2E). These alternative splicing patterns obtained from the NCBI Gene database were rechecked in the BLAT web page (https://genome.ucsc.edu). In summary, the *SAMD4A* C-form transcript exhibited a higher expression in the testis of the human compared to other isoforms, which was similar in the mouse, suggesting the conserved expression of the orthologous isoforms (*Samd4a* E-form and *SAMD4A* C-form) containing the SAM domain.

### Expression of murine *Samd4a* was specific to the spermatid stage

**FIGURE 2:**
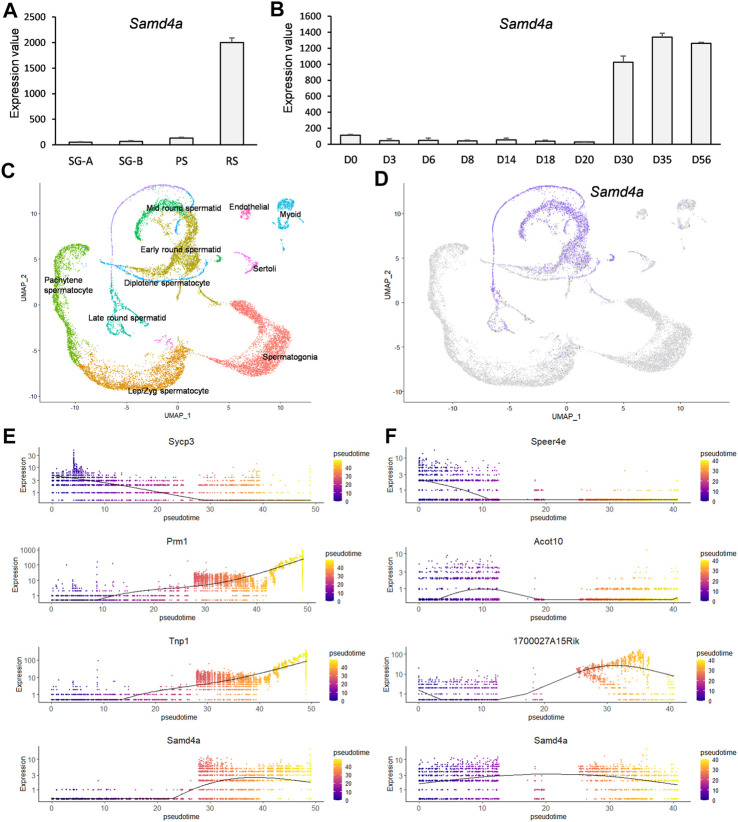
Developmental regulation of murine *Samd4a* expression. **(A)** The mRNA expression level of *Samd4a* in type A spermatogonia (SG-A), type B spermatogonia (SG-B), pachytene spermatocytes (PS), and round spermatids (RS) based on a microarray dataset (GSE4193). Values are presented as mean ± SEM. **(B)** The *Samd4a* mRNA expression during testis development (GSE926). The time course spans postpartum day 0 (D0) through day 56 (D56). Values represents the mean ± SEM. **(C)** Clustering of murine testicular cells visualized on a UMAP plot. For the dimensional reduction, raw single-cell RNA-seq data of testicular cells were retrieved under GSE109033. Each cluster was colored based on the expression of known marker genes. **(D)** Gene expression patterns of *Samd4a* in purple-colored cells on the UMAP projection. **(E)** The pseudotime analysis of spermatocyte- and spermatid-specific markers. *Sycp3* was used as a spermatocyte marker, and *Prm1* and *Tnp1* were employed as spermatid markers. Pseudotimes on the *x*-axis represent the relative progression each cell along the cellular development. **(F)** Pseudotime analysis of spermatid-specific markers. *Speer4e*, *Acot10*, and *1700027A15Rik* were used as early, mid, and late round spermatid markers, respectively. Expression of *Samd4a* in spermatids along the pseudotime is represented in the bottom of **(E)** and **(F)**.

To identify murine cell types in the testis which express *Samd4a*, expression values at multiple developmental stages were analyzed. Based on a microarray dataset (GSE4193), the *Samd4a* expression was substantially upregulated in round spermatids (Figure 2A). Also, according to GSE926, in the developing mouse testis, *Samd4a* expression was markedly increased in ages between 30 and 35 days postpartum (dpp) (Figure 2B) which correspond to a period before the formation of spermatozoa (mature sperm) begins around 35 dpp (Itman et al., 2006). In addition, a UMAP projection of integrated single-cell transcriptome revealed 10 clusters of murine testicular cells including germ cells and somatic cells (Figure 2C). Given the clusters, the expression of *Samd4a* was projected to be sequestered in early, mid-, and late round spermatids (Figure 2D). Furthermore, pseudotime profiles were examined for marker genes known to be expressed in spermatocytes (*Sycp3*) and spermatids (*Prm1* and *Tnp1*) (Hermann et al., 2018) (Figure 2E). According to cell density and regression lines, the expression pattern of *Samd4a* was similar to that of the spermatid markers which continued to increase along the pseudotime, but *Samd4a* expression slightly decreased from the late pseudotime around 40 after passing the plateau (Figure 2E). Last, compared to markers of early (*Speer4e*), mid- (*Acot10*), and late (*1700027A15Rik*) round spermatids (Hermann et al., 2018), the *Samd4a* expression tended to spread throughout the transition from early to late round spermatids (Figure 2F). Taken together, the expression of murine *Samd4a* was round spermatid-specific among the testicular cells.

### Spermatid-specific expression was common to the orthologous human *SAMD4A*

**FIGURE 3:**
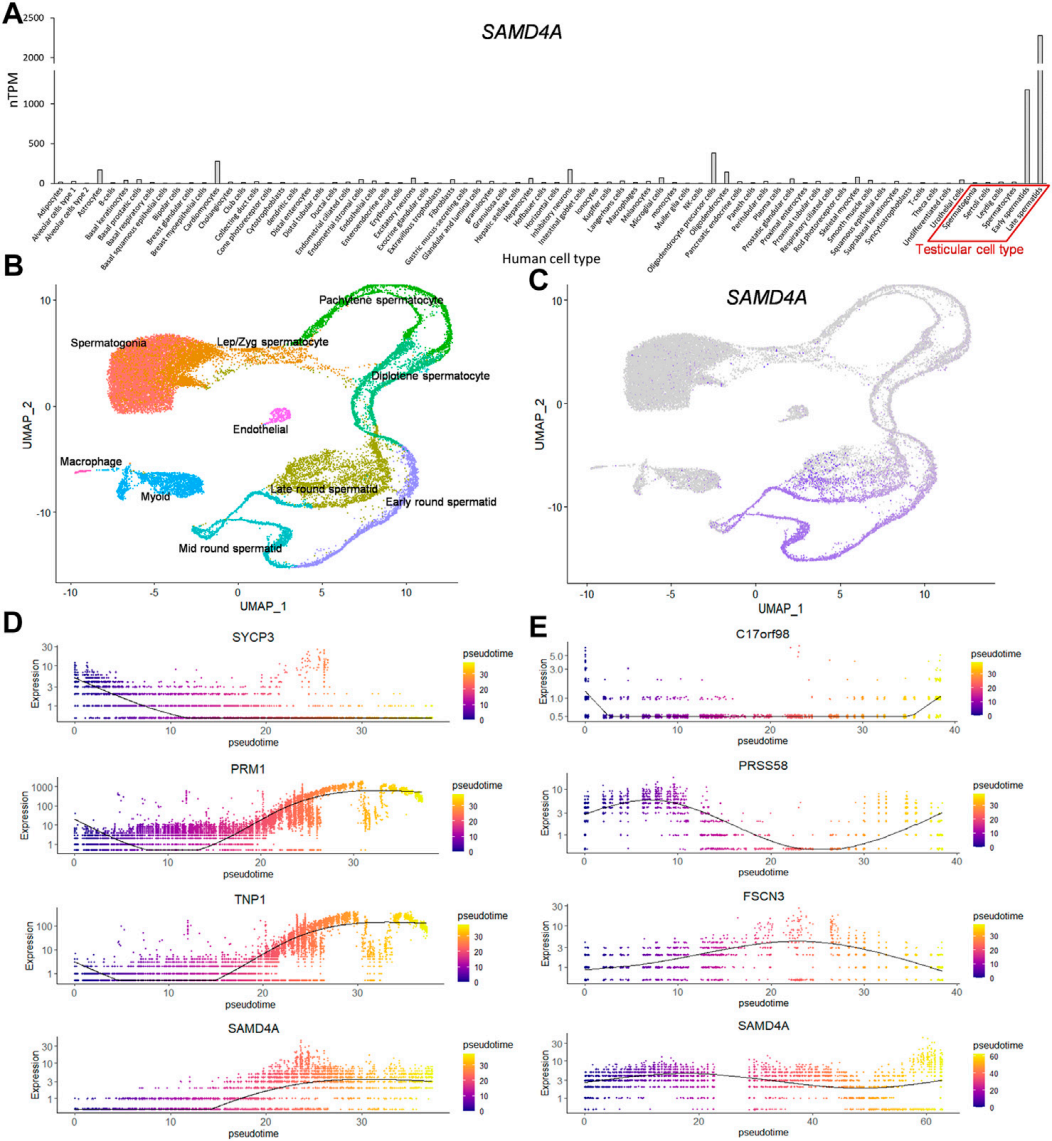
Developmental regulation of human *SAMD4A* expression. **(A)** The mRNA expression of *SAMD4A* in various cell types based on single cell RNA-seq data retrieved from the Human Protein Atlas (HPA) database. nTPM, normalized TPM. **(B)** A UMAP plot of human testicular cells grouped into 10 clusters based on scRNA-seq dataset (GSE109037) **(C)** Human *SAMD4A* expression patterns visualized in purple using UMAP projection. **(D)** Pseudotime analyses of hallmarks of human spermatocytes (*SYCP3*) and spermatids (*PRM1* and *TNP1*) along with human *SAMD4A*. **(E)** Expression of markers of early (*C17orf98*), mid (*PRSS58*), and late (*FSCN3*) round spermatids and the human *SAMD4A* gene along the pseudotime.

Comparison of *SAMD4A* expression values of various human cell types based on processed single-cell RNA-seq data from the Human Protein Atlas revealed that, among 76 analyzed cell types including testicular cells, the *SAMD4A* expression was markedly high in early and late spermatids (Figure 3A). To confirm the spermatid-specific expression of *SAMD4A*, single-cell RNA-seq data of human testicular cells were analyzed and, as a result, these cells were clustered into 10 groups in a UMAP plot (Figure 3B). As with the murine *Samd4a*, the expression of human *SAMD4A* was specific to early through late round spermatids (Figure 3C). Further pseudotime analyses revealed analogous expression patterns between the spermatid markers (*PRM1* and *TNP1*) and *SAMD4A*, compared to the spermatocyte marker (*SYCP3*) (Figure 3D). Also, unlike human spermatid stage-specific markers (*C17orf98* for early, *PRSS58* for mid, and *FSCN3* for late round spermatids) (Hermann et al., 2018), the *SAMD4A* expression covered early through late round spermatids (Figure 3E). Overall, the round spermatid-specific expression of human *SAMD4A* was similar to that of murine orthologous *Samd4a*, suggesting their conserved expression patterns.

### Generation of *Samd4a* knockout mice resulted in reduced testes sizes and weight and morphologically abnormal seminiferous tubules

**FIGURE 4:**
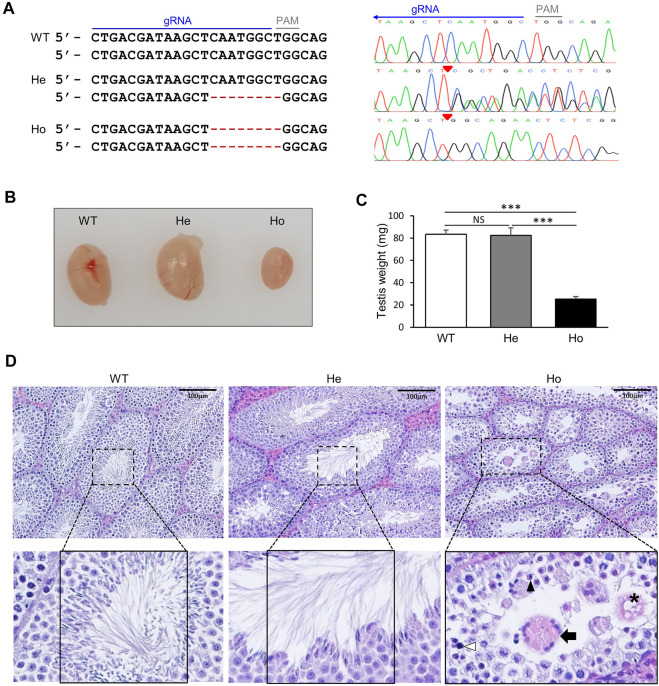
Generation of *Samd4a* knockout mice. **(A)** Nucleotide sequences of two alleles at exon two of *Samd4a* E form. A guide RNA sequence is underscored in blue. Red hyphens indicate deleted nucleotides derived from chromatograms. Red triangles on the chromatograms denote the beginning of deletion. PAM, protospacer adjacent motif. **(B)** Comparison of testis sizes and **(C)** weights. N = 5. ***, *p* < 0.001; *NS*, not significant. Values are represented as mean ± SEM. **(D)** Representative images of H&E staining of mouse testis. Seminiferous tubules are zoomed in. In the rumen of a seminiferous tubule of Ho, an apoptotic spermatid (black arrowhead), a multi-nucleated giant germ cell (black arrow), and degenerated germ cells (asterisk, a vacuolated/enlarged spermatid; white arrowhead, a pyknotic spermatid) were shown. WT, wild-type; He, heterozygous *Samd4a* knockout; Ho, homozygous *Samd4a* knockout.

By targeting exon two of testis-specific E-form of *Samd4a* using the CRISPR/Cas9 system, 8-nucleotide deletion was achieved, resulting in a frameshift mutation that created a premature stop codon before the SAM domain (Figure 4A). The sizes of testes in homozygous *Samd4a* knockouts (Ho) were substantially reduced compared to the wild-types (WT) and heterozygotes (He) (Figure 4B). The average weight of testes from Ho (25.16 mg ± 2.20) was approximately 3.3 times lighter than those of the WT (83.34 mg ± 3.52) and He (82.40 mg ± 6.01) (Figure 4C). Only the Ho knockouts exhibited shrinkage of seminiferous tubules (41,348μm^2^ ± 2817 for WT, 40,243μm^2^ ± 2019 for He, and 18,342μm^2^ ± 412 for Ho, Supplementary Figure S3) and abnormal morphologies of germ cells in the lumen within the seminiferous tubules (Figure 4D). Representative H&E-stained images further revealed that the lumen of seminiferous tubules of Ho knockout mice was filled with multi-nucleated giant germ cells (MGCs) and degenerated germ cells (DGs) including vacuolated or enlarged spermatids, pyknotic spermatids, and apoptotic spermatids with small nuclear fragments (Figure 4D). Also, unlike the WT and He knockout mice, spermatozoa were absent in the lumen of seminiferous tubules from the Ho knockout mice (Figure 4D). It suggested that *Samd4a* deficiency could lead to morphological abnormalities in the mouse testis.

### *Samd4a* deficiency impaired late stages of spermatogenesis

**FIGURE 5:**
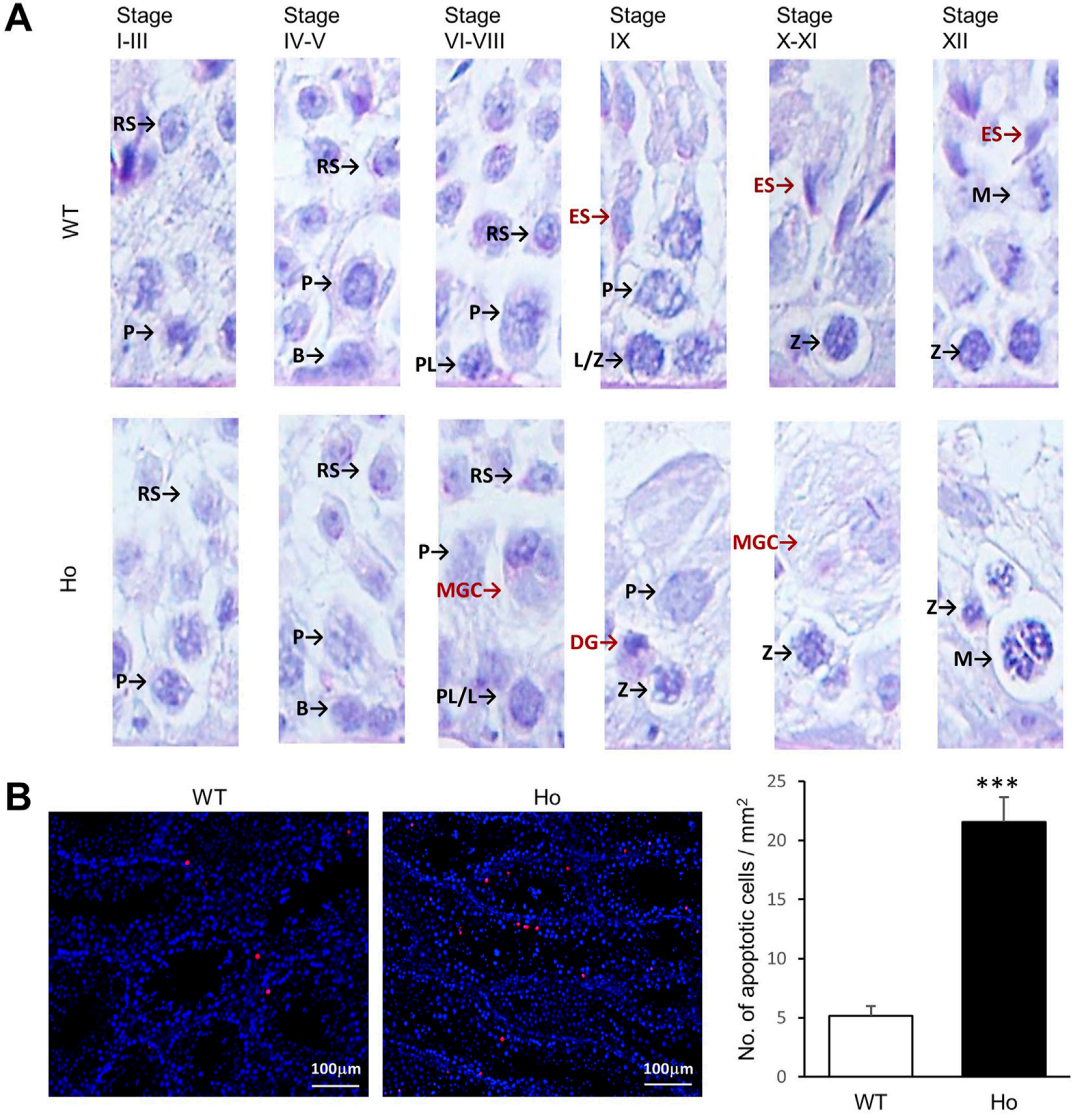
Abnormalities in the germ cells of *Samd4a* knockout mice. **(A)** Representative images of testicular cells at each developmental stage. RS, round spermatid; ES, elongated spermatid; *p*, pachytene spermatocyte; B, type B spermatogonia; PL, preleptotene spermatocyte; L, leptotene spermatocyte; Z, zygotene spermatocyte; M, meiotic spermatid; MGC, multi-nucleated giant germ cell; DG, degenerated germ cell. **(B)** Representative images of TUNEL apoptotic cell detection and the number of apoptotic cells within area. *N* = 5. ***, *p* < 0.001. Values represents mean ± SEM. WT, wild-type; Ho, homozygous *Samd4a* knockout.

Based on our findings on the absence of spermatozoa in homozygous *Samd4a* knockout mice, we further investigated which stages of spermatogenesis were affected by *Samd4a* deficiencies using PAS-stained testis sections. From the Stage I through V, it appeared that there were no differences in the types of germ cells from spermatogonia to round spermatids between the wild-type and homozygous knockout mice (Figure 5A). In Stages VI-VIII, MGCs appeared first in the homozygous knockout mice, implying the role of *Samd4a* at this stage. From Stage IX, elongated spermatids developed in the wild-type mice; whereas, round spermatids failed to develop into elongated spermatids in homozygous knockout mice. This absence of elongated spermatids in the homozygous knockout mice was maintained until Stage XII. During the stages without elongated spermatids in the testes of homozygous knockout mice, MGCs and DGs were observed unlike the wild-type mice. In addition, the TUNEL assay revealed that, in the homozygous *Samd4a* knockout mice, the number of apoptotic cells was significantly greater than that in the wild-type mice (*p* < 0.001) (Figure 5B). Taken together, it indicated that *Samd4a* deficiency in mice resulted in impairment of spermatogenesis, especially spermiogenesis due to abnormalities in testicular germ cells.

### The human *SAMD4A* expression was significantly less in non-obstructive azoospermia patients compared to individuals with obstructive azoospermia

**FIGURE 6:**
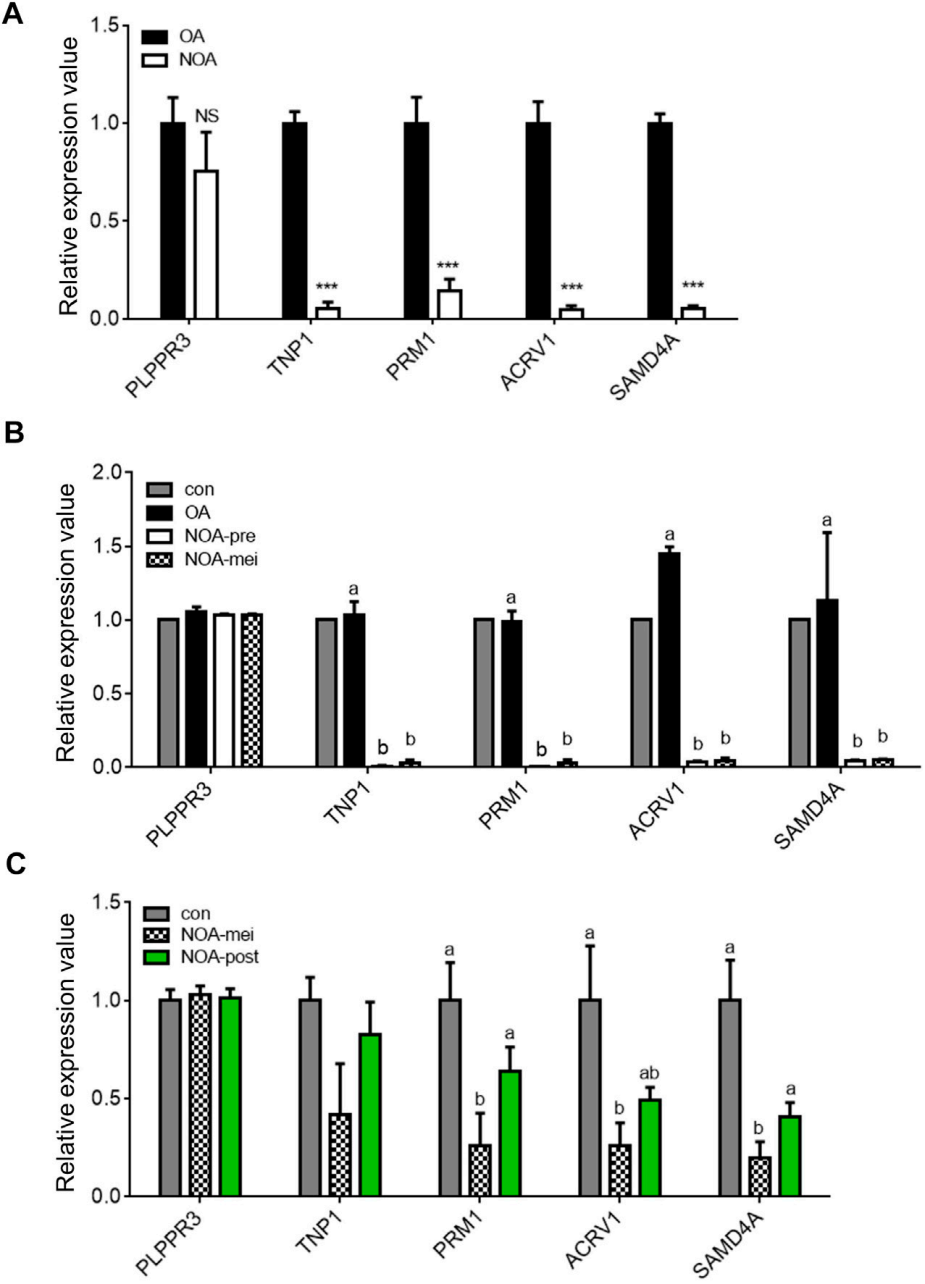
Profiling of *SAMD4A* expression in obstructive and nonobstructive azoospermia patients. **(A)** Comparisons of gene expression levels in testis samples between obstructive and nonobstructive azoospermia (OA and NOA, respectively) patients. The expression levels of stage-specific marker genes, *PLPPR3*, *TNP1*, *PRM1*, and *ACRV1*, in spermatogenesis and *SAMD4A* in testis samples of OA and NOA patients based on a microarray dataset (GSE145467). ***, *p* < 0.001. **(B)** Comparisons of gene expression levels in testis samples among pooled control (con), OA, nonobstructive azoospermia with pre-meiotic arrest (NOA-pre), and nonobstructive azoospermia with meiotic arrest (NOA-mei) by analyzing a microarray dataset (GSE108886). Letters, a and b, indicate a statistically significant difference. **(C)** Comparisons of gene expression levels in testis samples among normal (con), NOA-mei, and NOA arrested in post-meiotic stage (NOA-post) by analyzing a microarray dataset (GSE45885). Letters, a, ab and b, indicate a statistically significant difference. All values are presented as mean ± SEM.

Azoospermia characterized by no sperm in the ejaculate, is generally separated into two conditions, obstructive azoospermia (OA) and non-obstructive azoospermia (NOA). The distinction between OA and NOA is that normal spermatogenesis occurs in OA, but not in NOA. As the *SAMD4A* is mainly expressed in late-spermatogenic stages, it was investigated whether *SAMD4A* expression can reflect different types of cells at late stages of spermatogenesis between OA and NOA patients. Expression levels of *PLPPR3*, as a marker gene for spermatogonia stem cells (Sohni et al., 2019), were not different between OA and NOA patients. However, expression levels of spermatid marker genes (*TNP1* and *PRM1*) (Kleene, 2013; Hermann et al., 2018) and a sperm marker gene (*ACRV1*) (Foster et al., 1994) were significantly lower in the NOA than OA (Figure 6A). Also, *SAMD4A* expression was dramatically reduced in the testes of NOA patients compared to the OA (*p* < 0.001) (Figure 6A).

The condition of NOA is subcategorized into pre-meiotic arrest (NOA-pre), meiotic arrest (NOA-mei), and post-meiotic arrest (NOA-post). NOA-pre and NOA-mei patients have spermatogonia and/or spermatocytes but not spermatids, whereas, NOA-post patients have spermatogonia, spermatocytes, and spermatids in testes. The NOA-pre and NOA-mei conditions were compared using a dataset (GSE108886) and NOA-mei and NOA-post using another dataset (GSE45885). Although comparisons of NOA-pre and NOA-mei revealed that there was no expression difference in late stages of spermatogenesis (*TNP1*, *PRM1* and *ACRV1*), both groups have a significantly lower expression of those genes compared to the OA (Figure 6B). The *SAMD4A* expression was also significantly lower in testes of both NOA-pre and NOA-mei, compared to the OA (Figure 6B). Next, comparisons of NOA-mei and NOA-post revealed that NOA-mei showed lower expression levels of the late marker genes than those of NOA-post which were statistically comparable to the control levels (Figure 6C). Similarly, the expression level of *SAMD4A* was significantly higher in the NOA-post than the NOA-mei, but statistically comparable to the control (Figure 6C).

## Discussion

In this study, we report a testis-enriched gene, *Samd4a*, in mice that has yet to be investigated regarding spermatogenesis. The orthologous *SAMD4A* in the human also showed testis enrichment.

Our knockout mice studies revealed that *Samd4a* deficiency led to impaired spermatogenesis at late stages and subsequent abnormalities of testicular germ cells were observed, indicating potential roles of *Samd4a* in normal spermatogenesis. Our findings on the potential role of *Samd4a* during testicular development could be in line with the reported role of *Samd4a* on normal skeletogenesis in mice (Niu et al., 2017), in that transcriptional repression by SAMD4A could play a role in scavenging unwanted or inopportune gene transcripts during development. To begin with investigation at a transcript level, alternative splicing isoforms were compared and predominantly expressed transcripts in the testes of both mice and humans were identified. In both cases, the SAM domain-containing alternative splicing isoforms (*Samd4a* E-form in the mouse and *SAMD4A* C-form in the human) were predominantly expressed in the testis, implying the evolutionarily conserved roles of these isoforms. The SAM domain mediates binding of those isoforms to SRE-possessing mRNAs (Baez and Boccaccio, 2005). The RNA-binding protein genes encode two types of RNA-binding proteins: RNA-binding proteins with canonical RNA-binding domains (RBDs) such as the SAM domain and RNA-binding proteins without canonical RBDs (Aviv et al., 2003; Zhang et al., 2010; Zheng et al., 2021). In both cases, RNA-binding proteins are involved in post-transcriptional regulation *via* translational repression of mRNAs.

Previously, the *Drosophila* ortholog of *SAMD4A*, *Smaug*, has been reported to hinder translation initiation by preventing recruitment of translation initiation factors (Nelson et al., 2004), but the mechanism of translation repression in mice and humans remains to be elucidated. As recently reported in the case of *SAMD4A* knockdown in osteosarcoma epithelial cells (Fernandez-Alvarez et al., 2022), defects in the formation of *SAMD4A*-mediated proteinaceous MLOs sequestering mRNAs lead to failures in mRNA repression and a release of unnecessary mRNAs that triggers inopportune protein translation. Therefore, investigations on testicular developmental stages in which *SAMD4A* can mediate RNA binding is of great importance. The developmental gene expression patterns of *Samd4a* showed spermatid-specific expression, suggesting that RNA binding of *SAMD4A* and repression of target mRNAs containing SRE occur during the spermatid stages (Figures 2A,D). Also, the expression of *Samd4a* during development of the mouse testis was markedly increased prior to spermatozoa formation around 35 dpp (Itman et al., 2006) (Figure 2B). These patterns were highly conserved in the expression of the human orthologous *SAMD4A*, implying cross-species functional relationships between SAMD4A proteins (Figure 3). In particular, in comparison with marker genes for spermatocytes (*Sycp3*/*SYCP3*) and spermatids (*Prm1*/*PRM1* and *Tnp1*/*TNP1*), murine spermatid stage-specific markers [early (*Speer4e*), mid (*Acot10*), and late (*1700027A15Rik*) round spermatids], and human spermatid stage-specific markers [early (*C17orf98*), mid (*PRSS58*), and late (*FSCN3*) round spermatids] (Hermann et al., 2018), the pseudotime analysis showed that *Samd4a*/*SAMD4A* expression tended to be fairly consistent across the early/mid/late round spermatid stages, indicating that *Samd4a*/*SAMD4A* is a suitable marker for spermatids.

Although male infertility was reported in chemically induced-homozygous mutation of *Samd4a* (Chen et al., 2014), there was no actual data presented to support this. Our current study using knockout mice proved that the homozygous knockout of *Samd4a* led to abnormal germ cell morphology which was observed in almost all seminiferous tubules and shrinkage of testicular tubules (Figure 4 and Supplementary Figure S3). A previous study reported that disruption of RNA-binding protein can cause male infertility with increased apoptotic cells in testis (Zheng et al., 2021). In this study, increased numbers of apoptotic cells including pyknotic spermatids found in the seminiferous epithelium of *Samd4a* ^−/−^ mice suggest that disruption of *Samd4a* can cause spermatogenic failure. Along with these defects, absence of elongated spermatids in *Samd4a* knockout testis further suggests that *Samd4a* is essential for normal spermatogenesis.

Azoospermia causing male infertility can be categorized into OA and NOA, comprising about 40% and 60%, respectively (The management of infertility due to obstructive azoospermia, 2008). Because obstruction in the ductal system is the cause of OA (The management of infertility due to obstructive azoospermia, 2008), OA patients have mature sperm as in the testes of a normal male (named as the control in this study). In the current study, similar expression levels of spermatogenic markers and *SAMD4A* between the control and OA samples further confirmed normal spermatogenesis in testes of OA patients. However, expression levels of genes involved in mid- and late stages of spermatogenesis [spermatid marker genes (*TNP1* and *PRM1*) and a sperm marker gene (*ACRV1*)] and *SAMD4A* were significantly lower in the NOA patients compared to the OA, unlike the expression level of *PLPPR3*, a marker gene for spermatogonia stem cells (Sohni et al., 2019), which was not different between OA and NOA patients. These expression data and *Samd4a* KO mice showing impairment of late stages of spermatogenesis that are characteristics of NOA patients suggest *SAMD4A* as a potential genetic factor associated with the NOA.

Generally, the NOA can be subcategorized into the NOA-pre and NOA-mei, which exhibit presence of spermatogonia and/or spermatocytes only, and the NOA-post, which exhibits presence of elongated spermatids. Our analyses using microarray data sets revealed that the marker genes in mid- and late spermatogenesis (*TNP1*, *PRM1*, and *ACRV1*) were comparably expressed between the NOA-pre and NOA-mei testis samples due possibly to absence of spermatids in both groups. However, the mid- and late spermatogenic marker genes and *SAMD4A* were more expressed in the NOA-post compared to the NOA-mei due possibly to presence and absence of spermatids, respectively. These data further support that *SAMD4A* is the spermatid specific marker that can serve as a new genetic marker for the NOA-post among other NOA patient groups.

Taken together, the current study clearly proved that *Samd4a/SAMD4A* genes and their splicing isoforms are testis-specific and spermatid-enriched. To the best of our knowledge, this study is the first investigation that revealed degenerative effects of *Samd4a* knockout in mice on testicular germ cells and spermatogenesis. Furthermore, this study provides spermatid-specific SAMD4A as a candidate biomarker for categorizing subgroups of non-obstructive azoospermia patients.

## Data Availability

The datasets presented in this study can be found in online repositories. The names of the repository/repositories and accession number(s) can be found in the article/Supplementary Table S3.
