# Supplementary material for: A novel testis-enriched gene, Samd4a, regulates spermatogenesis as a spermatid-specific factor
Source: Front Cell Dev Biol. 2022 Oct 5;10:978343. doi: 10.3389/fcell.2022.978343 (PMC9579339; doi:10.3389/fcell.2022.978343)
Supplement: Supplementary file 1 [file Presentation1.PPTX]

## Slide 1
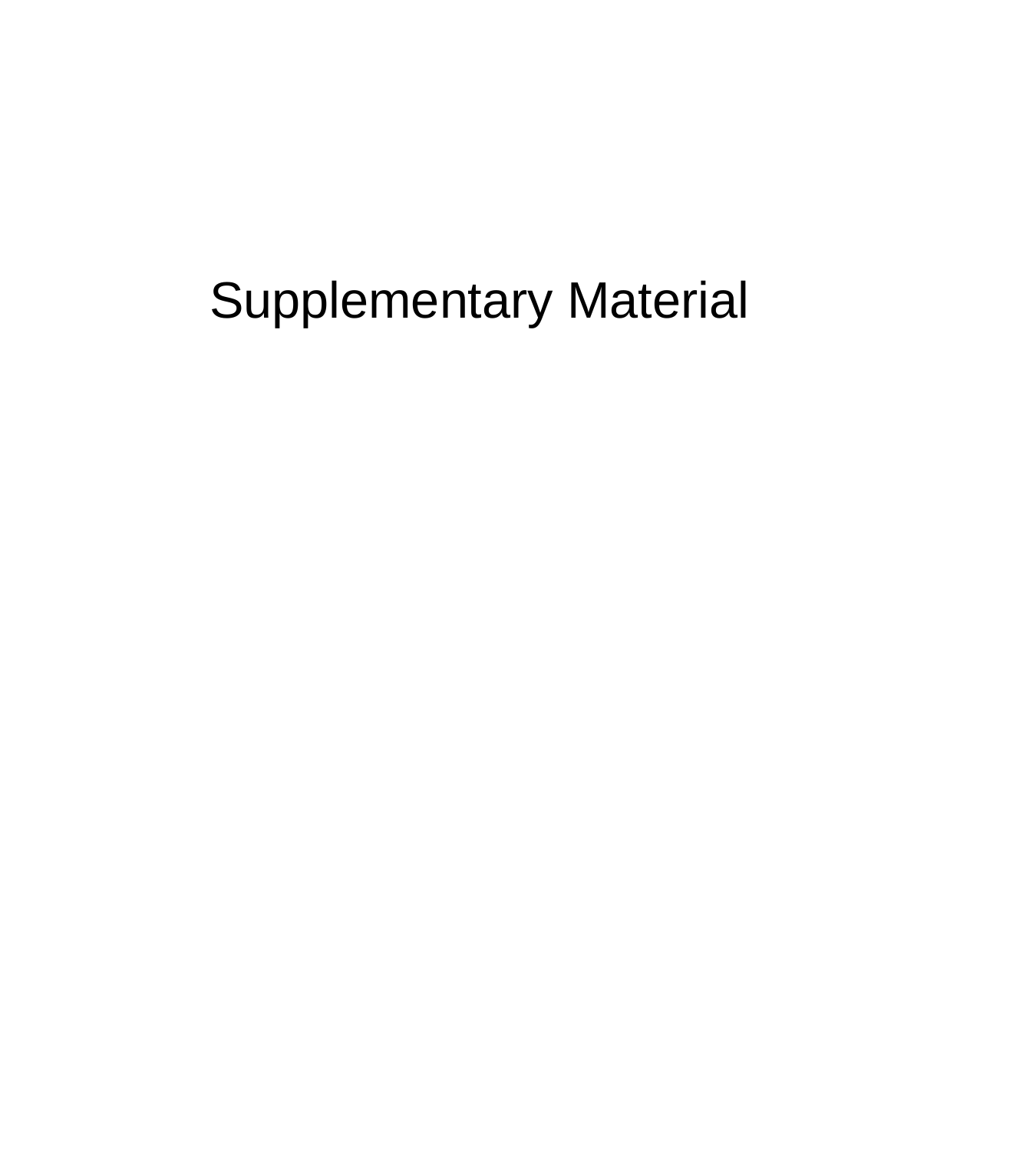

# Supplementary Material

## Slide 2
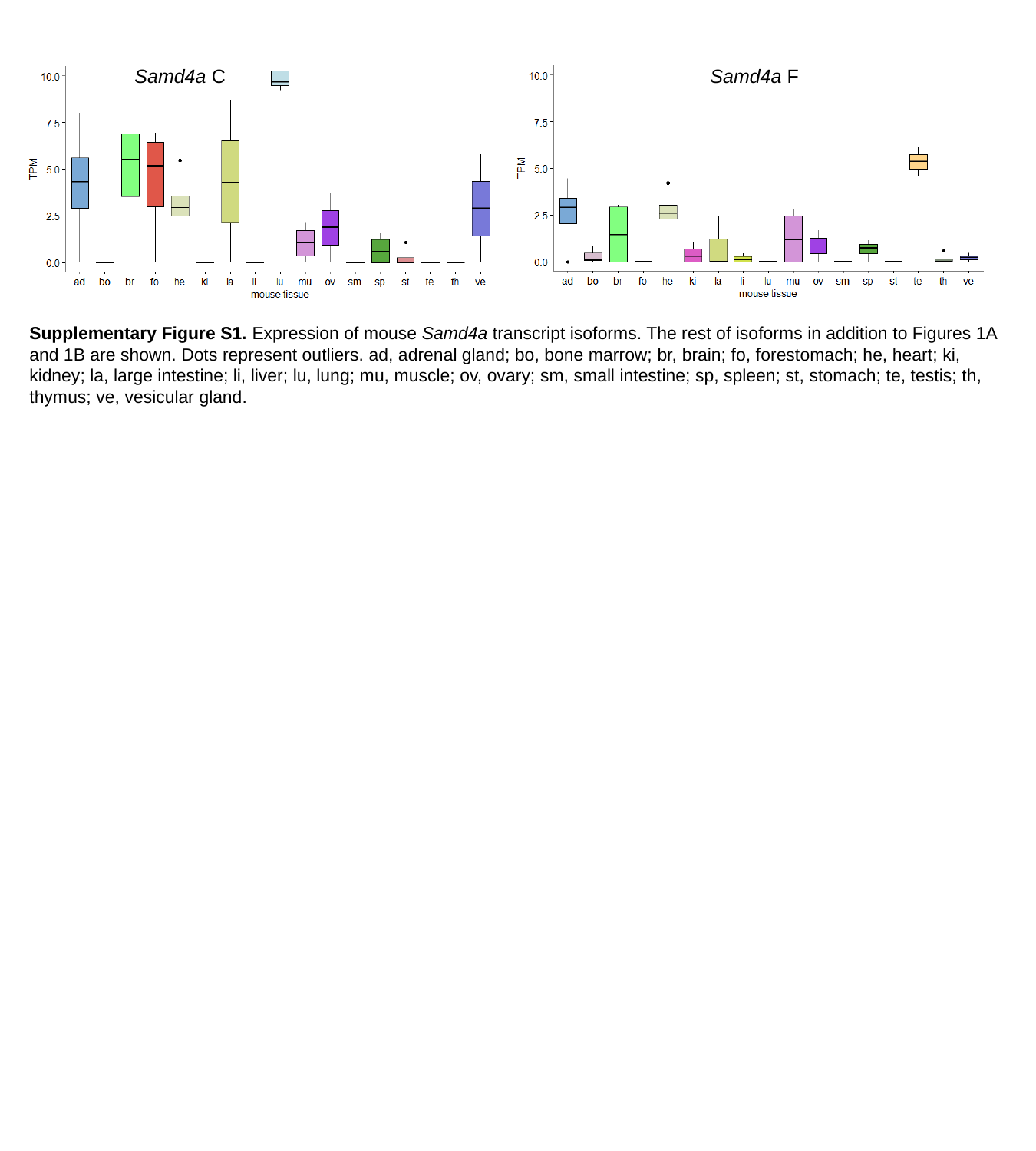

Samd4a C
Samd4a F
Supplementary Figure S1. Expression of mouse Samd4a transcript isoforms. The rest of isoforms in addition to Figures 1A and 1B are shown. Dots represent outliers. ad, adrenal gland; bo, bone marrow; br, brain; fo, forestomach; he, heart; ki, kidney; la, large intestine; li, liver; lu, lung; mu, muscle; ov, ovary; sm, small intestine; sp, spleen; st, stomach; te, testis; th, thymus; ve, vesicular gland.

## Slide 3
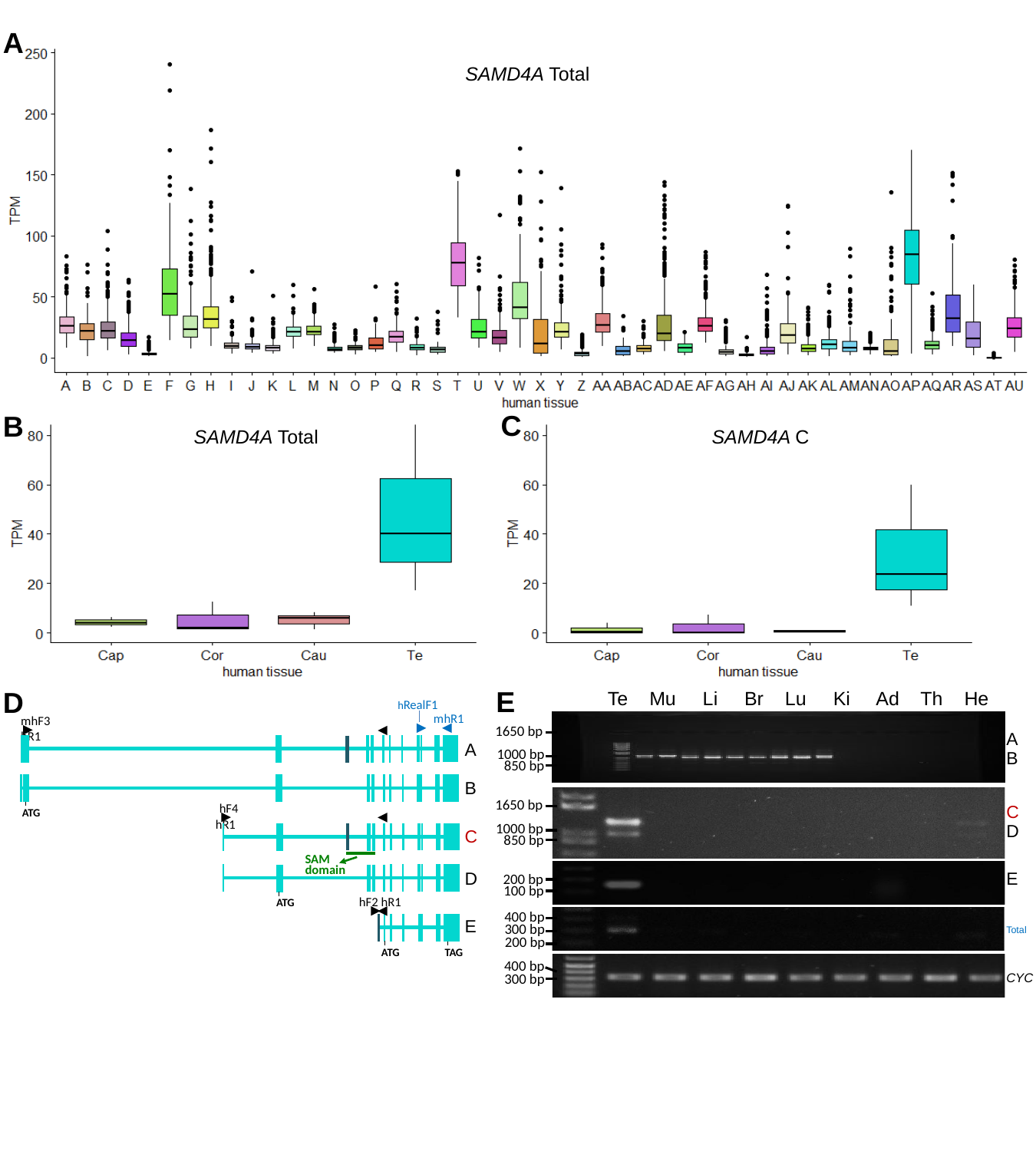

A
B
D
SAMD4A Total
C
SAMD4A Total
SAMD4A C
E
Te Mu Li Br Lu Ki Ad Th He
1650 bp
1000 bp
850 bp
1650 bp
1000 bp
850 bp
200 bp
100 bp
400 bp
300 bp
200 bp
400 bp
300 bp
 hRealF1
 mhR1
mhF3 hR1
A
B
C
D
E
Total
CYC
A
B
C
D
E
 hF4 hR1
ATG
SAM
domain
 hF2 hR1
ATG
ATG
TAG

## Slide 4
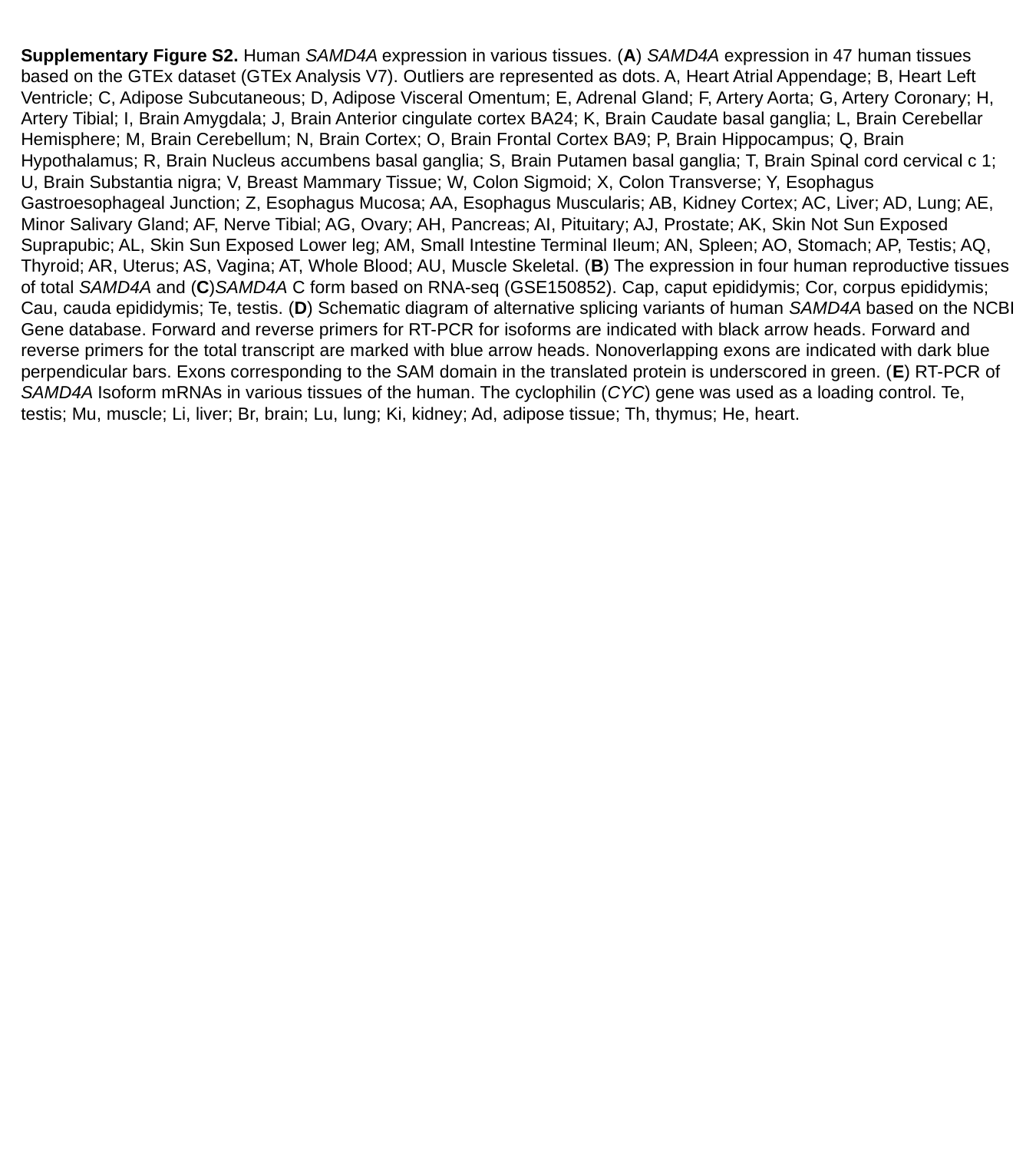

Supplementary Figure S2. Human SAMD4A expression in various tissues. (A) SAMD4A expression in 47 human tissues based on the GTEx dataset (GTEx Analysis V7). Outliers are represented as dots. A, Heart Atrial Appendage; B, Heart Left Ventricle; C, Adipose Subcutaneous; D, Adipose Visceral Omentum; E, Adrenal Gland; F, Artery Aorta; G, Artery Coronary; H, Artery Tibial; I, Brain Amygdala; J, Brain Anterior cingulate cortex BA24; K, Brain Caudate basal ganglia; L, Brain Cerebellar Hemisphere; M, Brain Cerebellum; N, Brain Cortex; O, Brain Frontal Cortex BA9; P, Brain Hippocampus; Q, Brain Hypothalamus; R, Brain Nucleus accumbens basal ganglia; S, Brain Putamen basal ganglia; T, Brain Spinal cord cervical c 1; U, Brain Substantia nigra; V, Breast Mammary Tissue; W, Colon Sigmoid; X, Colon Transverse; Y, Esophagus Gastroesophageal Junction; Z, Esophagus Mucosa; AA, Esophagus Muscularis; AB, Kidney Cortex; AC, Liver; AD, Lung; AE, Minor Salivary Gland; AF, Nerve Tibial; AG, Ovary; AH, Pancreas; AI, Pituitary; AJ, Prostate; AK, Skin Not Sun Exposed Suprapubic; AL, Skin Sun Exposed Lower leg; AM, Small Intestine Terminal Ileum; AN, Spleen; AO, Stomach; AP, Testis; AQ, Thyroid; AR, Uterus; AS, Vagina; AT, Whole Blood; AU, Muscle Skeletal. (B) The expression in four human reproductive tissues of total SAMD4A and (C)SAMD4A C form based on RNA-seq (GSE150852). Cap, caput epididymis; Cor, corpus epididymis; Cau, cauda epididymis; Te, testis. (D) Schematic diagram of alternative splicing variants of human SAMD4A based on the NCBI Gene database. Forward and reverse primers for RT-PCR for isoforms are indicated with black arrow heads. Forward and reverse primers for the total transcript are marked with blue arrow heads. Nonoverlapping exons are indicated with dark blue perpendicular bars. Exons corresponding to the SAM domain in the translated protein is underscored in green. (E) RT-PCR of SAMD4A Isoform mRNAs in various tissues of the human. The cyclophilin (CYC) gene was used as a loading control. Te, testis; Mu, muscle; Li, liver; Br, brain; Lu, lung; Ki, kidney; Ad, adipose tissue; Th, thymus; He, heart.

## Slide 5
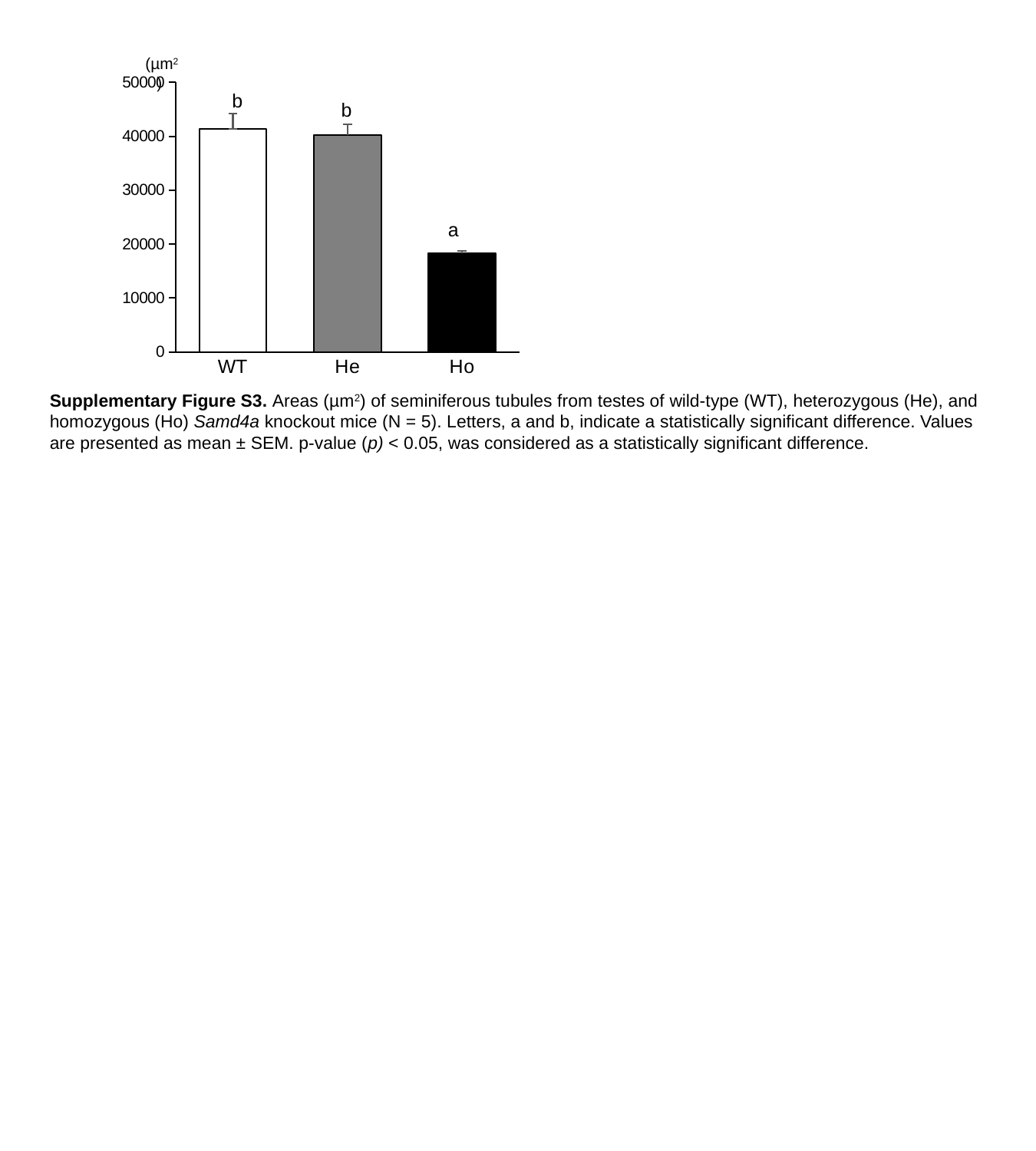

(µm2)
### Chart
| Category | |
|---|---|
| WT | 41348.06328 |
| He | 40191.01433333333 |
| Ho | 18342.738410750364 |b
b
a
Supplementary Figure S3. Areas (µm2) of seminiferous tubules from testes of wild-type (WT), heterozygous (He), and homozygous (Ho) Samd4a knockout mice (N = 5). Letters, a and b, indicate a statistically significant difference. Values are presented as mean ± SEM. p-value (p) < 0.05, was considered as a statistically significant difference.

## Slide 6
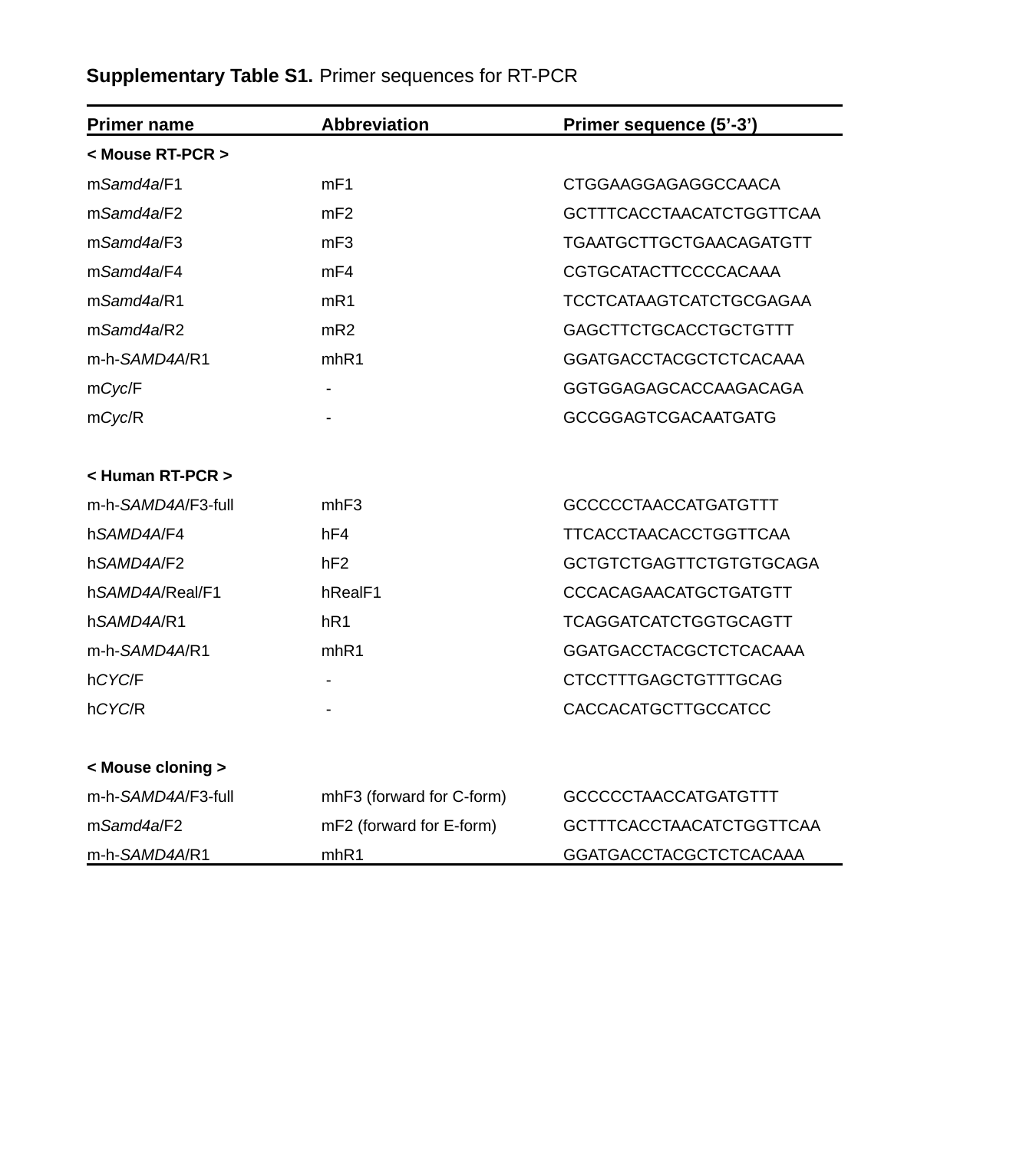

Supplementary Table S1. Primer sequences for RT-PCR
| Primer name | Abbreviation | Primer sequence (5’-3’) |
| --- | --- | --- |
| < Mouse RT-PCR > | | |
| mSamd4a/F1 | mF1 | CTGGAAGGAGAGGCCAACA |
| mSamd4a/F2 | mF2 | GCTTTCACCTAACATCTGGTTCAA |
| mSamd4a/F3 | mF3 | TGAATGCTTGCTGAACAGATGTT |
| mSamd4a/F4 | mF4 | CGTGCATACTTCCCCACAAA |
| mSamd4a/R1 | mR1 | TCCTCATAAGTCATCTGCGAGAA |
| mSamd4a/R2 | mR2 | GAGCTTCTGCACCTGCTGTTT |
| m-h-SAMD4A/R1 | mhR1 | GGATGACCTACGCTCTCACAAA |
| mCyc/F | - | GGTGGAGAGCACCAAGACAGA |
| mCyc/R | - | GCCGGAGTCGACAATGATG |
| | | |
| < Human RT-PCR > | | |
| m-h-SAMD4A/F3-full | mhF3 | GCCCCCTAACCATGATGTTT |
| hSAMD4A/F4 | hF4 | TTCACCTAACACCTGGTTCAA |
| hSAMD4A/F2 | hF2 | GCTGTCTGAGTTCTGTGTGCAGA |
| hSAMD4A/Real/F1 | hRealF1 | CCCACAGAACATGCTGATGTT |
| hSAMD4A/R1 | hR1 | TCAGGATCATCTGGTGCAGTT |
| m-h-SAMD4A/R1 | mhR1 | GGATGACCTACGCTCTCACAAA |
| hCYC/F | - | CTCCTTTGAGCTGTTTGCAG |
| hCYC/R | - | CACCACATGCTTGCCATCC |
| | | |
| < Mouse cloning > | | |
| m-h-SAMD4A/F3-full | mhF3 (forward for C-form) | GCCCCCTAACCATGATGTTT |
| mSamd4a/F2 | mF2 (forward for E-form) | GCTTTCACCTAACATCTGGTTCAA |
| m-h-SAMD4A/R1 | mhR1 | GGATGACCTACGCTCTCACAAA |

## Slide 7
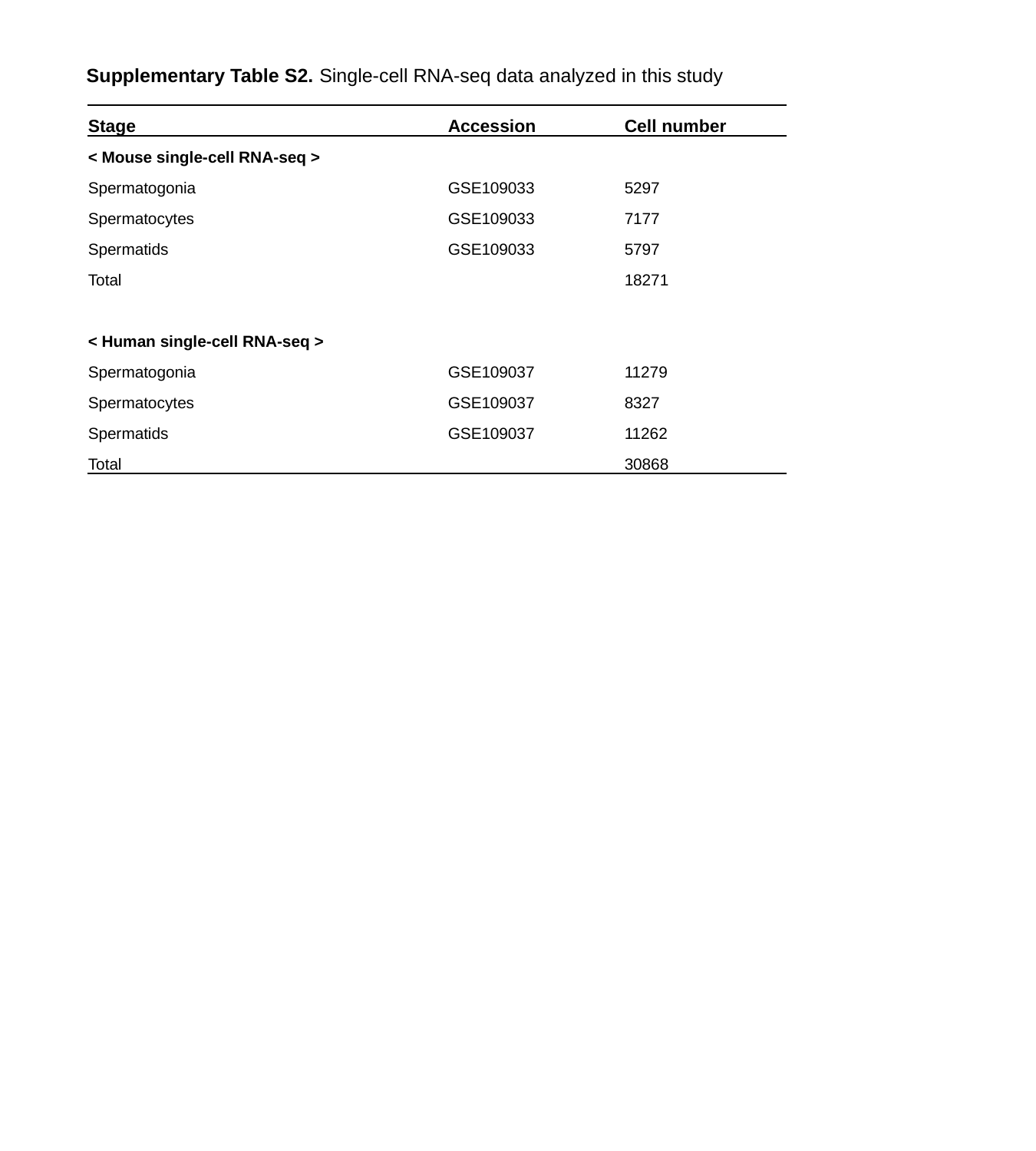

Supplementary Table S2. Single-cell RNA-seq data analyzed in this study
| Stage | Accession | Cell number |
| --- | --- | --- |
| < Mouse single-cell RNA-seq > | | |
| Spermatogonia | GSE109033 | 5297 |
| Spermatocytes | GSE109033 | 7177 |
| Spermatids | GSE109033 | 5797 |
| Total | | 18271 |
| | | |
| < Human single-cell RNA-seq > | | |
| Spermatogonia | GSE109037 | 11279 |
| Spermatocytes | GSE109037 | 8327 |
| Spermatids | GSE109037 | 11262 |
| Total | | 30868 |

## Slide 8
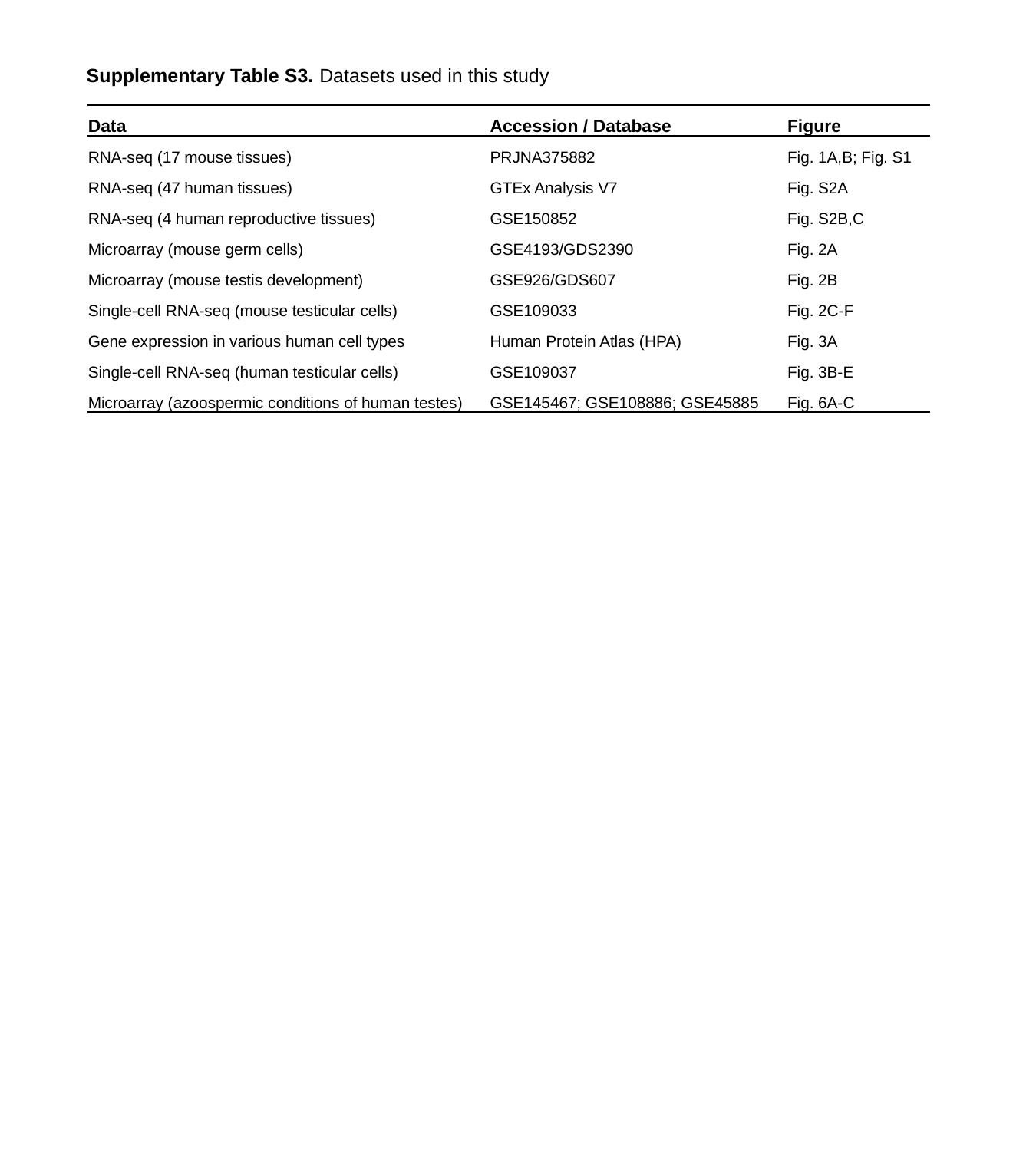

Supplementary Table S3. Datasets used in this study
| Data | Accession / Database | Figure |
| --- | --- | --- |
| RNA-seq (17 mouse tissues) | PRJNA375882 | Fig. 1A,B; Fig. S1 |
| RNA-seq (47 human tissues) | GTEx Analysis V7 | Fig. S2A |
| RNA-seq (4 human reproductive tissues) | GSE150852 | Fig. S2B,C |
| Microarray (mouse germ cells) | GSE4193/GDS2390 | Fig. 2A |
| Microarray (mouse testis development) | GSE926/GDS607 | Fig. 2B |
| Single-cell RNA-seq (mouse testicular cells) | GSE109033 | Fig. 2C-F |
| Gene expression in various human cell types | Human Protein Atlas (HPA) | Fig. 3A |
| Single-cell RNA-seq (human testicular cells) | GSE109037 | Fig. 3B-E |
| Microarray (azoospermic conditions of human testes) | GSE145467; GSE108886; GSE45885 | Fig. 6A-C |
